# Mitogen-activated protein kinase activator with WD40 repeats (MAWD) and MAWD-binding protein induce cell differentiation in gastric cancer

**DOI:** 10.1186/s12885-015-1637-7

**Published:** 2015-09-15

**Authors:** Dongmei Li, Jun Zhang, Yu Xi, Lei Zhang, Wenmei Li, Jiantao Cui, Rui Xing, Yuanmin Pan, Zemin Pan, Feng Li, Youyong Lu

**Affiliations:** 1 Department of Biochemistry and Molecular Biology, School of Medicine, Shihezi University, Xinjiang, 832000 P.R. China; 2 Laboratory of Molecular Oncology, Key Laboratory of Carcinogenesis and Translational Research (Ministry of Education), Peking University Cancer Hospital/Institute, Beijing, 100142 P.R. China; 3 Beijing Genomics Institute, Chinese Academy of Sciences, Shunyi, Beijing, 101318 P.R. China; 4 Department of General Surgery, First Affiliated Hospital of Shihezi University, Shihezi, Xinjiang 832008 P.R. China; 5 Department of Laboratory, First Affiliated Hospital of Shihezi University, Shihezi, Xinjiang 832008 P.R China

## Abstract

**Background:**

Our previous proteomic analysis revealed that mitogen-activated protein kinase activator with WD40 repeats (MAWD) and MAWD-binding protein (MAWBP) were downregulated in gastric cancer (GC) tissues. These proteins interacted and formed complexes in GC cells. To investigate the role of MAWD and MAWBP in GC differentiation, we analyzed the relationship between MAWD/MAWBP and clinicopathologic characteristics of GC tissues and examined the expression of E-cadherin and pepsinogen C (PGC)—used as gastric mucosa differentiation markers—in MAWD/MAWBP-overexpressing GC cells and xenografts.

**Methods:**

We measured MAWD, MAWBP, transforming growth factor-beta (TGF-beta), E-cadherin, and PGC expression in 223 GC tissues and matched-adjacent normal tissues using tissue microarray and immunohistochemistry (IHC) analyses, and correlated these expression levels with clinicopathologic features. MAWD and MAWBP were overexpressed alone or together in SGC7901 cells and then E-cadherin, N-cadherin, PGC, Snail, and p-Smad2 levels were determined using western blotting, semiquantitative RT-PCR, and immunofluorescence analysis. Alkaline phosphatase (AKP) activity was measured to investigate the differentiation level of various transfected cells, and the transfected cells were used in tumorigenicity assays and for IHC analysis of protein expression in xenografts.

**Results:**

MAWD/MAWBP positive staining was significantly lower in GC tissues than in normal samples (*P* < 0.001), and the expression of these proteins was closely correlated with GC differentiation grade. Kaplan–Meier survival curves indicated that low MAWD and MAWBP expression was associated with poor patient survival (*P* < 0.05). The differentiation-related proteins E-cadherin and PGC were expressed in GC tissues at a lower level than in normal tissues (*P* < 0.001), but were upregulated in MAWD/MAWBP-overexpressing cells. N-cadherin and Snail expression was strongr in vector-expressing cells and comparatively weaker in MAWD/MAWBP co-overexpressing cells. MAWD/MAWBP co-overexpression inhibited Smad2 phosphorylation and nuclear translocation (*P* < 0.05), and AKP activity was lowest in MAWD/MAWBP coexpressing cells and highest in vector-expressing cells (*P* < 0.001). TGF-beta, E-cadherin, and PGC expression in xenograft tumors derived from MAWD/MAWBP coexpressing cells was higher than that in control.

**Conclusions:**

MAWD and MAWBP were downregulated and associated with the differentiation grade in GC tissues. MAWD and MAWBP might induce the expression of differentiation-related proteins by modulating TGF-beta signaling in GC cells.

**Electronic supplementary material:**

The online version of this article (doi:10.1186/s12885-015-1637-7) contains supplementary material, which is available to authorized users.

## Background

Gastric cancer (GC) is one of the most common malignancies worldwide and ranks second in terms of global cancer-related mortality [1]. Host genetic factors as well as bacterial virulence, environmental, and several other factors have been shown to affect the gastric oncogenic process, but the underlying molecular mechanism is poorly understood.

GC displays distinct biological behaviors according to histological differentiation [2, 3], and the prognosis of GC patients is closely associated with histological classification: The 5-year survival rates of GC patients are 90 %, 50 %–60 %, and 10 %–15 % for GC Stages I, II, and III, respectively [4]. Thus, it is critical to elucidate the regulatory mechanism of GC cell differentiation, and previous studies have investigated the mechanism of induced differentiation in GC cells. Sakamoto *et al.* determined that in addition to intestinal transcription factor caudal type homeobox 2, epidermal growth factor receptor (EGFR) activation induces LI-cadherin expression and participates in the intestinal differentiation in GC [5]. Wei *et al.* reported that P27 regulation by glycogen synthase kinase-3beta results in hexamethylene bisacetamide-induced differentiation of human GC cells [6]. Hsu *et al.* found that the loss of RUNX3 expression correlates with GC differentiation [7]. However, few reports have been published on proteins related to the differentiation and proliferation of GC cells.

Previously, we determined—using 2D gel electrophoresis and mass spectrometry—that the expression of mitogen-activated protein kinase activator with WD40 repeats (MAWD) and MAWD-binding protein (MAWBP) was markedly attenuated in GC tissues. These proteins interacted and formed complexes in GC cells, and this might play a major role in GC carcinogenesis [8].

The effects of MAWD in cancers have been described in a few reports. MAWD is evolutionarily conserved and expressed in diverse tissues [9, 10]. Iriyama and colleagues attempted to detect MAWD-related proteins by using the conventional two-hybrid technique and found that MAWBP can bind to MAWD [10]. Buess *et al.* reported complete or partial allelic loss of MAWD in 45.2 % (75/166) of colorectal cancers [11]. Jung *et al.* found that MAWD bound to NM23-H1 and that this created a complex that interacted with, and potentiated the activity of, p53 [12]. Dong *et al.* detected chromosomal deletions in prostate cancer that overlapped with the *MAWD* location [13]. Matsuda *et al.* determined that MAWD was overexpressed in 45.6 % (21/46) of human breast tumor tissues and promoted anchorage-independent cell growth [9]. Kim *et al.* reported MAWD upregulation in 50.8 % (30/59) of adenomas and 70.7 % (87/123) of colorectal cancers [14]. Lastly, Halder *et al.* found that serine-threonine kinase receptor-associated protein, or STRAP, was upregulated in 60 % (12/20) of colon and 78 % (11/14) of lung carcinomas [15]. However, no reports have been published on the function of MAWD in GC, and little is known about MAWBP other than that it can interact with MAWD.

MAWD, as the name suggests, contains a WD40 repeat domain [16]. Datta *et al.* showed that MAWD recruits Smad7 and forms a complex that increases the inhibition of transforming growth factor-beta (TGF-beta) signaling [17, 18]. We hypothesized that MAWD and MAWBP interactions play a key role in the differentiation of GC. Therefore, we investigated the relationship between the expression of MAWD/MAWBP and the differentiation grade of GC by using clinical samples, and we also examined the expression of differentiation-related proteins in MAWD/MAWBP-overexpressing GC cells and xenografts. Lastly, we determined whether MAWD and MAWBP induce differentiation through TGF-beta signaling in GC. Research on proteins that influence the differentiation of GC will not only contribute to the diagnosis of GC: it will also help guide GC treatment.

## Methods

### Sample collection

Clinical data and GC samples were collected from Beijing Cancer Hospital of Peking University, Beijing, China, from January 2011 to June 2013. None of the patients received chemotherapy or radiotherapy before tissue samples were obtained. All histological diagnoses were confirmed by experienced pathologists at the hospital. Written informed consent was obtained from all patients regarding the use of the collected samples in research studies. The patient records and information were anonymized and de-identified before analysis. The research project and the informed consent were examined and certified by the Ethics Committee of the School of Oncology, Peking University (Beijing Cancer Hospital, China) (No. ECBCH-2011228).

### Immunohistochemistry (IHC) and tissue microarray (TMA)

The gastric TMA was constructed using a tissue arraying instrument (Beecher Instruments, Silver Spring, USA), as described previously [19]. The avidin-biotin-peroxidase protocol was used for IHC. The antibodies used were against MAWBP (1:100; custom-made, clone number AbM51007) and MAWD (1:300; custom-made, clone number AbP61014) [8], and TGF-beta (1:100; cat# ab66043, Abcam, Cambridge, UK), E-cadherin (1:100; cat# 610182, BD, Franklin, USA), and pepsinogen C (PGC) (1:150; cat# R31924, Sigma, Cambridge, USA). Samples were incubated with antibodies at 4 °C overnight and visualized using the DAB kit (Dako, Glostrup, Denmark). All sections were examined and scored by 2 pathologists in a blinded evaluation. Staining was scored based on intensity and proportion. The signal intensity was scored as 0, no staining; 1+, low intensity; 2+, moderate intensity; or 3+, high intensity. The extent of surface area containing the target protein was scored on a scale of 0–3: (0,: no staining; 1+: present, but <20 %; 2+: 20 %–50 %; and 3+: >50 %). The positivity score was calculated by multiplying staining intensity and surface area data by tissue compartment (range: 0–9), and the composite scores were separated using a four-tier system (negative: 0–1; 1+: 2–4; 2+: 5–7; and 3+: 8–9).

### Prediction for potential MAWD and MAWBP protein-protein interaction (PPI) networks

The PPI network provides an integrative view of molecular processes. The human protein interaction network was retrieved from http://www.hprd.org/; MAWD- and MAWBP- interacting proteins were then searched for candidate protein-interaction sequence motifs (trimers and tetramers).

### Plasmid construction

We reconstructed MAWD and MAWBP expression vectors using pcDNA3.1 B (−). Total RNA was extracted from 19-week-old fetal liver. MAWD and MAWBP cDNAs were produced using reverse-transcription PCR (RT-PCR). The MAWD primers were the following: forward: 5’-CGCGGATCCATGGCAATGAGACA GACG-3’, reverse: 5’-CCCAAGCTTTCAGGCCTTAACATCAGG-3’. The amplicons were 1053 bp in size. The MAWBP primers were the following: forward: 5’- AACTTGGTCG ACCAGCTTGCAAGGAAAATG-3’, reverse: 5’-ATAACTCGAGCTAGGCTGTCAGTGT GCC-3’. The amplicons were 867 bp in size. PCR was performed as follows: the reaction was initiated using a 5-min incubation at 94 °C, and this was followed by 35 cycles of 94 °C for 45 s, 56 °C for 45 s, and 72 °C for 60 s, and then the reaction was terminated after a 10-min extension at 72 °C. Products were purified through gel extraction, and the recombinant plasmids were transferred into *Escherichia coli* DH5α and then identified by performing restriction-enzyme digestion and sequencing analysis.

### Cell culture and transfection

The cell line SGC7901 was routinely maintained as previously described [20]. SGC7901 cells were selected and cultured at 60 %–70 % confluence in 35-mm plates and then transfected with recombinant MAWD and MAWBP plasmids or empty vector by using Lipofectamine 2000 (Invitrogen, Carlsbad, CA, USA). MAWD and MAWBP plasmids were co-transfected into SGC7901 cells, and at 48 h post-transfection, the cells were seeded in selection medium containing 400 μg/mL G418 and cultured for 21 days to screen for stable clones.

### RT-PCR and western blotting

To confirm efficient transfection, RT-PCR and western blotting were performed. Total RNA was extracted using Trizol (Invitrogen) and 5 μg of the RNA was reverse transcribed and PCR-amplified. The primers used and the amplicon sizes were the following: MAWD: forward, 5’-GGGACAGGATAAACTTTAGC-3’, and reverse, 5’-AGCATGATCCCAAAGTCG AAC-3’ (amplicon size, 162 bp); and MAWBP: forward, 5’-GGGTCTGCACACGCTGTTC-3’, and reverse: 5’-TAATGTCAACCCTTCCGTCT-3 (132 bp). The internal control, beta-actin, was processed concurrently with all specimens. The other primers used were the following: E-cadherin: forward, 5’-TGATTCTGCTGCTCTTGCTG-3’, and reverse, 5’-CTCTTCTCCGCC TCCTTCTT-3’ (122 bp); N-cadherin: forward, 5’-CGTG AAGGTTTGCCAGTGT-3’, and reverse, 5’- CAGCACAAGGATAAGCAGGA-3’ (130 bp); PGC: forward: 5’-CGTCC ACCTACTCCACCAAT-3’, and reverse, 5’-CACTCAA GCCGAACTCCTG-3’(132 bp); and Snail: forward, 5’-CCAGAGTTTACCTTCCAGCA G-3’, and reverse, 5’-GACA GAGTCCCAGATGAGCA-3’ (214 bp). All primers were synthesized by Sangon Biotech (Shanghai) Co., Ltd. (Shanghai, China).

For western blotting, cell extracts were prepared and then the proteins (50 μg) were separated on 12 % SDS-PAGE and transferred to PVDF membranes. The blots were stained (overnight, 4 °C) with the following antibodies (diluted in blocking buffer): anti-MAWD (1:500), anti-MAWBP (1:500), anti-Snail (1:1000; cat# C15D3, Cell Signaling, Danvers, USA), anti-E-cadherin (1:1000), anti-N-cadherin (1:1000; cat# 610921, BD), anti-PGC (1:1000), and anti-p-Smad2 (1:500; cat# AB3849, Millipore, Temecula, USA). The immunoreactive bands were detected using Super Signal West Dura Extended Duration Substrate (Thermo Scientific, Rockford, USA). These experiments were repeated thrice.

### Immunofluorescence

Cells were grown on glass slides, washed with PBS, methanol-fixed for 10 min, and then processed for immunofluorescence. Cells were exposed to antibodies against E-cadherin, N-cadherin, Snail, PGC, and p-Smad2 (all diluted 1:50) overnight at 4 °C, and then incubated for 60 min with fluorophore-conjugated secondary antibodies; nuclei were stained with 4′,6-diamidino-2-phenylindole (DAPI). Cells were examined using a Confocal Fluorescence Imaging Microscope TCS-SP5 (Leica, Mannheim, Germany). Three repeated scan results of mean fluorescence intensity were analyzed.

### Alkaline phosphatase (AKP) assay

The Alkaline Phosphatase Assay Kit (Jiancheng Bioengineering Institute, Nanjing, China) was used for measuring intracellular AKP activity. We used 3 hree holes for the detection and repeated this test thrice. Washed cells (1 × 10^6^) were homogenized in assay buffer, resuspended in 500 μL of PBS, and then lysed through ultrasonication. Assay and reaction buffers were added to 5 μL of cell lysates and incubated for 15 min at 37 °C, and then 150 μL of the color development reagent was added and mixed. Absorbance was measured at 520 nm using an iMark Microplate Reader.

### Tumorigenicity assay in nude mice

Transfected cells were washed twice and resuspended in 1× Hank’s buffer at a concentration of 5 × 10^6^ cells/mL. A 100-μL cell suspension was then injected subcutaneously into the left dorsal flank of 15 5-week-old female nude mice; the right side was inoculated with SGC7901 cells transfected with vector alone and this served as the control. The larger (*a*) and smaller (*b*) tumor diameters were measured every week, and tumor volume was calculated as *a* × *b*^2^ × 0.5. At 3.5 weeks after injections, the mice were anesthetized with high-concentration diethyl ether until they died. Tumor specimens were split and collected. RT-PCR (described above) was used to analyze MAWD and MAWBP expression, and IHC analysis was used for detecting MAWBP, MAWD, TGF-beta, E-cadherin, and PGC protein expression. All animal procedures were approved by the Ethics Committee of the School of Oncology, Peking University (Beijing Cancer Hospital, China).

### Statistical analysis

Statistical analyses were performed using Statistic Package for Social Science (SPSS) version 16.0. The *χ*^2^ test was used to define significant differences and univariate analysis among the pathological samples. *P* < 0.05 was considered statistically significant. The Spearman rho test was performed to evaluate the protein correlations. The Kaplan–Meier method was used for predicting patient overall survival according to levels of MAWD and MAWBP expression. Student’s *t* test was used in measurement data.

## Results

### Characterization of MAWD and MAWBP coexpression and clinical outcome in gastric tumor

**Fig. 1:**
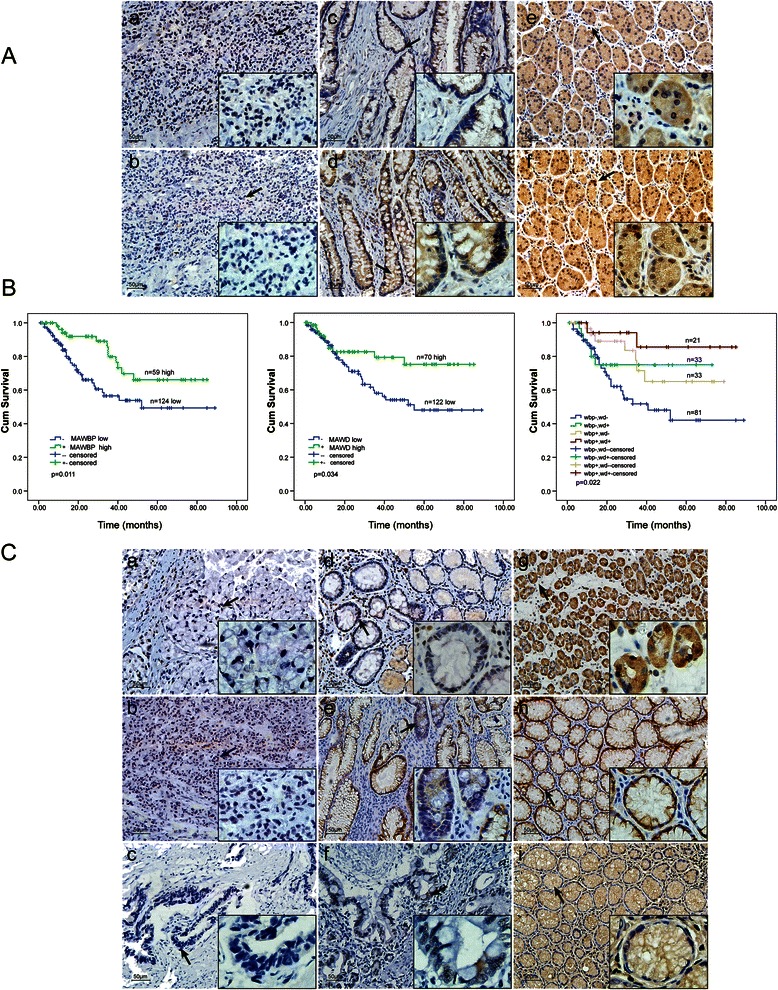
Comparison of MAWBP, MAWD, TGF-beta, E-cadherin, and PGC expression in GC and normal tissues by using IHC. (**a**) Comparison of MAWBP and MAWD expression in GC and normal tissues by means of TMA and IHC analysis (100×; 400× in the lower right corner). Weak MAWBP (a) and MAWD (b) protein staining in poorly differentiated carcinoma; expression of MAWBP (c) and MAWD (d) in intestinal metaplasia; strong positive staining of MAWBP (e) and MAWD (f) in normal tissues (*P* < 0.001). (**b**) Kaplan–Meier analysis of overall survival in GC patients expressing different levels of MAWBP and MAWD. (a) Green and blue lines represent the survival curves of patients expressing high and low levels of MAWBP (*P* < 0.05). (b) Green and blue lines represent the survival curves of patients expressing MAWD at high and low levels (*P* < 0.05). (c) Combined MAWBP and MAWD expression for analysis of overall survival; prognosis was better for patients who expressed high levels of MAWBP and MAWD than for patients who expressed the proteins at low levels (*P* < 0.05). (**c**) Comparison of TGF-beta, E-cadherin, and PGC expression in GC and normal tissues by using TMA and IHC analysis (100×; 400× in the lower right corner). Weak TGF-beta (a), E-cadherin (b), and PGC (c) protein staining in poorly differentiated carcinoma; staining for TGF-beta (d), E-cadherin (e), and PGC (f) in intestinal metaplasia; strong positive staining for TGF-beta (g), E-cadherin (h), and PGC (i) in normal tissues (*P* < 0.05)

We compared the expression levels of MAWD and MAWBP proteins in the TMA that contained 223 GC samples and adjacent normal tissues. GC tissues showed faint or negative MAWD and MAWBP expression. Representative IHC staining is shown in Fig. 1a. The rate of positive MAWD expression in gastric tumor tissues was 75/223 (32.2 %), which was lower than that in normal samples (51/86; 59.3 %) (Table 1). MAWBP showed the same expression pattern as MAWD did. The positivity rate of MAWBP in gastric tumor tissues was 62/223 (26.6 %), whereas it was 50/81 (61.7 %) in normal tissues (Table 1) (Fig. 1a). MAWD and MAWBP expression displayed statistically significant correlation (*P* < 0.001) (Table 2).

**Table 1:** Comparison of MAWBP, MAWD, TGF-beta, E-cadherin, and PGC protein expression in GC and normal tissues

	Expression		
Protein	Tumor	Normal	*P-*value
	(% positive)	(% positive)	
MAWBP	62/223 (26.6)	50/81 (61.7)	<0.001
MAWD	75/223 (32.2)	51/86 (59.3)	<0.001
TGF-beta	105/223 (47.1)	54/87 (62.1)	0.012
E-cadherin	95/223 (42.6)	66/95 (69.5)	<0.001
PGC	86/223 (38.6)	72/100 (72)	<0.001

**Table 2:** Correlation of MAWBP and MAWD expression in GC

	Expression	MAWBP		
Protein		Positive (%)	Negative (%)	*P-*value
MAWD	Positive	32/215 (14.9)	42/215 (19.5)	0.001
	Negative	26/215 (12.1)	115/215 (53.5)	

Further examination of the samples revealed that well-differentiated cancers tended to show uniform MAWD and MAWBP expression. The Kaplan–Meier survival curve indicated that prognosis was better for patients who expressed MAWD and MAWBP at high levels than for patients who expressed the proteins at low levels (*P* < 0.05) (Fig. 1b). These data suggest that analysis of the expression of both MAWD and MAWBP should provide useful information and might enhance the identification of differentiation grade and prognosis in patients.

### Correlation of TGF-beta, E-cadherin, and PGC protein expression with MAWD and MAWBP in GC tumors

Given the clear relationship between MAWD and MAWBP expression and differentiation and the correlation of their expression with TGF-beta signaling, we performed TMA analysis for TGF-beta, E-cadherin, and PGC, which are GC differentiation-related proteins (Fig. 1c). The positive staining rates for these differentiation-related proteins in tumor and normal tissues were, respectively, the following (Table 1): TGF-beta, 105/223 (47.1 %) and 54/87 (62.1 %) (*P* < 0.05); E-cadherin, 95/223 (42.6 %) and 66/95 (69.5 %) (*P* < 0.001); and PGC, 86/223 (38.6 %) and 72/100 (72 %) (*P* < 0.001).

Relationship analysis revealed that MAWD and MAWBP expression was significantly correlated with the expression of TGF-beta (*P* < 0.001) and E-cadherin (*P* < 0.05) (Tables 3, 4). The results of Spearman rho test indicated the expression levels of MAWBP, MAWD, TGF-beta, and E-cadherin were correlated with each other (*P* < 0.05), and that the expression of E-cadherin was correlated with that of PGC (*P* < 0.001) (Table 5). Table 6 presents a summary of our analysis of patient clinicopathologic characteristics in relation to the expression level of each of the aforementioned proteins.

**Table 3:** Correlation of MAWD expression with TGF-beta, E-cadherin, and PGC expression in GC

	Expression	MAWD		
Protein		Positive (%)	Negative (%)	*P-*value
TGF-beta	Positive	49/209 (23.4)	52/209 (24.9)	<0.001
	Negative	24/209 (11.5)	84/209 (40.2)	
E-cadherin	Positive	37/200 (18.5)	49/208 (23.6)	0.039
	Negative	33/200 (13.9)	81/200 (40.5)	
PGC	Positive	35/218 (16.5)	48/218 (22)	0.45
	Negative	39/218 (17.9)	96/218 (44)

**Table 4:** Correlation of MAWBP expression with TGF-beta, E-cadherin, and PGC expression in GC

	Expression	MAWBP		
Protein		Positive (%)	Negative (%)	*P-*value
TGF-beta	Positive	43/216 (19.9)	59/216 (27.3)	<0.001
	Negative	13/216 (6.0)	101/216 (46.8)	
E-cadherin	Positive	31/208 (14.9	58/208 (27.9)	0.026
	Negative	25/208 (15.7)	94/208 (45.2)	
PGC	Positive	28/223 (12.0)	57/223 (25.6)	0.112
	Negative	32/223 (14.3)	106/223 (47.5)

**Table 5:** Correlations among the expression patterns of 5 proteins in GC

Correlation coefficient (ρ), *N* = 223					
	MAWBP	MAWD	TGF-beta	E-cadherin	PGC
MAWBP	1	0.266**	0.350**	0.160*	0.107
MAWD		1	0.267**	0.146*	0.136*
TGF-beta			1	0.161*	0.048
E-cadherin				1	0.403**
PGC					1

*P < 0.05, **P < 0.001

**Table 6:** Univariate analysis with clinicopathological features in GC

Features	MAWBP expression (%)	MAWD expression (%)	TGF-beta expression (%)	E-cadherin expression (%)	PGC expression (%)
Sex					
Male	37/151(24.5)	44/146(30.1)	69/149(46.3)	60/143(41.9)	54/152(35.5)
Female	22/71(30.9)	25/68(36.8)	29/65(44.6)	35/64(54.6)	36/72(50.0)
*P-*value	0.195	0.209	0.469	0.061	0.028
Age at diagnosis					
<60	24/91(26.3)	36/89(40.4)	41/88(46.6)	38/83(45.8)	36/91(38.8)
≥60	35/128(27.3)	31/121(29.2)	55/122(45.1)	54/121(44.6)	52/129(38.9)
*P-*value	0.500	0.017	0.469	0.492	0.512
TNM stage					
I	12/20(60.0)	9/20(45.0)	11/20(55.0)	11/20(50.0)	10/21(47.6)
II	18/78(17.2)	24/75(32.0)	31/75(41.3)	28/72(38.9)	32/78(41.0)
III	11/64(17.2)	14/60(23.3)	27/61(44.3)	32/61(52.5)	29/65(44.6)
IV	18/54(33.3)	21/52(40.3)	27/51(52.9)	21/48(43.8)	17/53(32.1)
*P-*value	0.001	0.160	0.501	0.355	0.481
Tumor depth					
T1-T2	16/45(36)	19/46(41.3)	25/45(55.6)	17/42(40.5)	18/47(38.3)
T3-T4	43/173(24.9)	49/163(30.1)	72/164(43.9)	75/161(46.6)	70/172(40.7)
*P-*value	0.107	0.105	0.111	0.298	0.451
Lymph node status					
N0	21/52(40.4)	20/52(38.5)	27/51(52.9)	24/47(51.1)	23/54(42.6)
N1	18/77(23.4)	24/74(32.4)	32/73(43.8)	29/77(37.7)	36/78(46.2)
N3	12/68(17.6)	18/62(29.0)	29/66(43.9)	31/62(50.0)	23/66(34.8)
N4	8/19(42.1)	6/19(31.6)	9/17(52.9)	8/15(53.3)	6/19(31.6)
*P-*value	0.016	0.760	0.681	0.339	0.452
Distant metastasis					
M0	51/190(26.8)	56/183(30.6)	81/182(44.5)	82/179(45.8)	78/192(40.6)
M1	8/28(28.6)	12/26(46.2)	16/27(59.3)	10/24(41.7)	10/27(37.0)
*P-*value	0.504	0.089	0.110	0.437	0.446
Differentiation					
D1	6/12(50.0)	4/11(36.3)	9/12(75.0)	8/12(66.7)	1/13(7.6)
D2	30/78(38.5)	28/76(36.8)	41/74(55.4)	36/71(50.7)	32/79(40.5)
D3	22/123(17.9)	35/120(29.2)	45/120(37.5)	47/116(40.5)	52/125(41.6)
*P-*value	0.031	0.649	0.006	0.046	0.035

### Overexpression of MAWD and MAWBP in GC cells

**Fig. 2:**
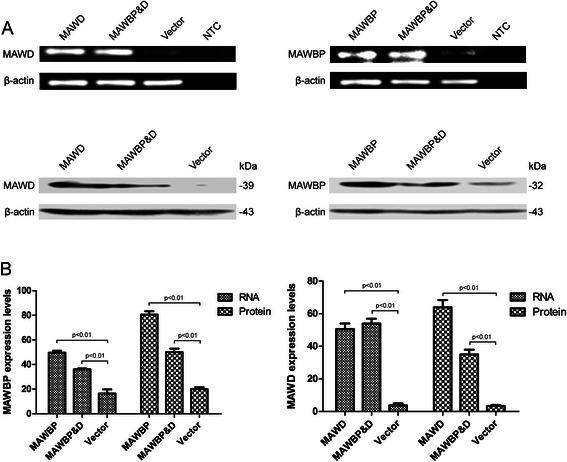
Stable overexpression of MAWBP and MAWD in the GC cell line SGC7901. (**a**) Expression of MAWBP and MAWD was detected in stable clones by means of RT-PCR and western blotting. (**b**) The mRNA and protein levels of MAWBP and MAWD were higher in stable clones than in control cells (*P* < 0.001)

Previously, we detected endogenous expression of MAWD and MAWBP in GC cell lines using real-time PCR and western blotting. We found that MAWD and MAWBP are expressed at low levels in SGC7901 cells [21]. Thus, we selected SGC7901 as the test cell line and transfected the cells with the MAWD and MAWBP eukaryotic expression vectors that we constructed; the cells were transfected with each of the vectors alone or with both vectors. We named these groups of cells MAWD (overexpressing MAWD alone), MAWBP (overexpressing MAWBP alone), MAWBP&D (co-overexpressing MAWBP and MAWD), and Vector. Next, we isolated G418-resistant clones in order to obtain cells that stably overexpressed the proteins, and we used RT-PCR and western blotting to check for efficient expression of MAWD and MAWBP (*P* < 0.001; Fig. 2a, b).

### MAWD and MAWBP coexpression induces differentiation in GC cells

**Fig. 3:**
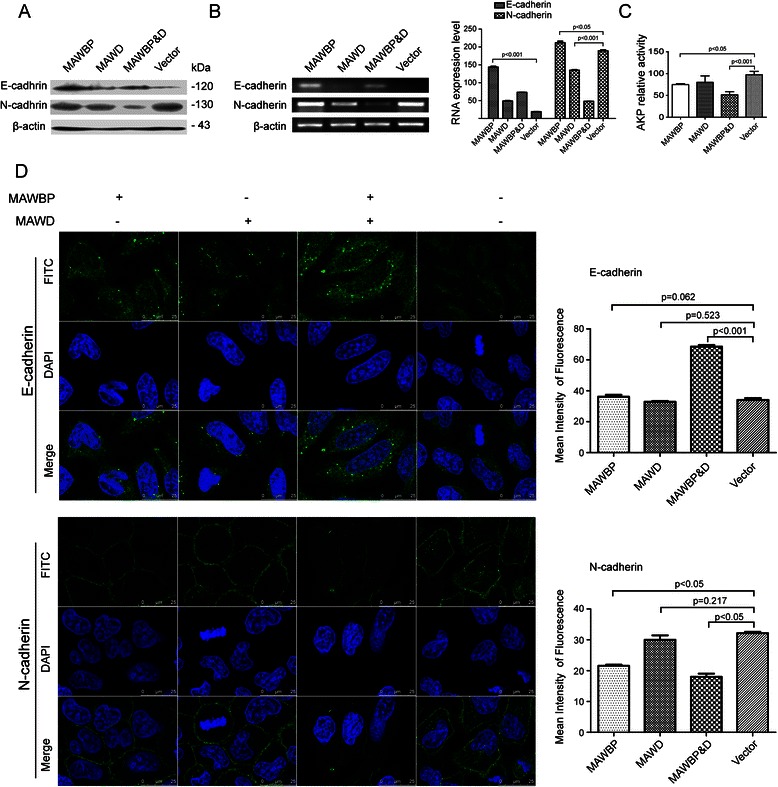
Expression of E-cadherin and N-cadherin in GC cells overexpressing MAWBP and MAWD. (**a**) E-cadherin and N-cadherin protein levels were measured through western blotting. E-cadherin expression was increased relative to control in the MAWBP and MAWBP&D groups and was weakest in the Vector group, and N-cadherin levels were decreased in the MAWBP&D group. (**b**) E-cadherin and N-cadherin mRNA levels were estimated using semiquantitative RT-PCR. E-cadherin expression was again elevated in the MAWBP and MAWBP&D groups and weakest in the Vector group, and N-cadherin expression was decreased in the MAWBP&D group. (**c**) AKP activity measurements revealed that the AKP level was lowest in the MAWBP&D group and highest in the control group (*P* < 0.05). (**d**) E-cadherin and N-cadherin protein expression was analyzed using confocal microscopy. The mean fluorescence intensity shows that E-cadherin expression was increased in the MAWBP&D group (*P* < 0.001) and N-cadherin expression was elevated in the Vector group (*P* < 0.05). The cells in the MAWBP&D group were morphologically well organized and appeared to exhibit polarity, whereas the cells in the control group were disorganized

**Fig. 4:**
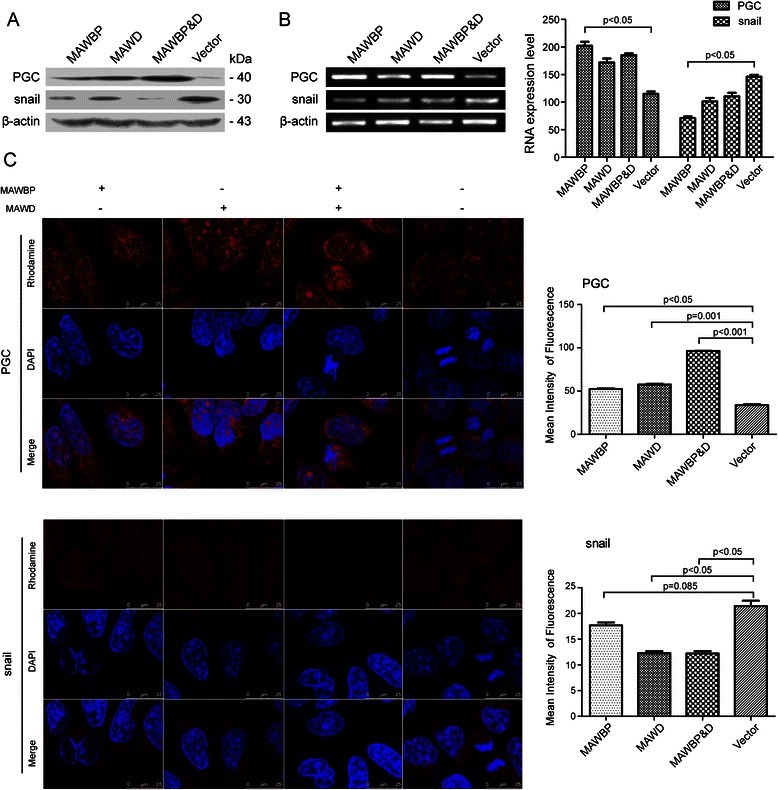
Expression of PGC and Snail in GC cells overexpressing MAWBP and MAWD. (**a**) PGC and Snail protein levels were measured through western blotting. PGC expression was highest in the MAWBP&D group (*P* < 0.001) and lowest in the Vector group, whereas Snail expression was weakest in the MAWBP&D group (*P* < 0.001). (**b**) PGC and Snail mRNA levels were estimated using semiquantitative RT-PCR. PGC expression was lowest in the Vector group, but N-cadherin expression was highest in the Vector group (*P* < 0.05). (**c**) PGC and Snail protein expression levels were analyzed using a confocal microscope. The mean fluorescence intensity shows that PGC expression was strongest in the MAWBP&D group (*P* < 0.001) and Snail expression was strongest in the Vector group (*P* < 0.05). Once again, the cells in the MAWBP&D group were morphologically well organized, but the Vector-group cells were disorganized

We performed western blotting, semiquantitative RT-PCR, and confocal microscopy in order to examine the expression of the differentiation-related proteins E-cadherin, PGC, N-cadherin, and Snail in transfected cells. E-cadherin and PGC were used as differentiation markers of the gastric mucosa. The expression of E-cadherin protein and mRNA was increased relative to control in the MAWBP&D group and was weakest in the Vector group (Fig. 3a, b), and this was also shown by the results of confocal microscopy and mean fluorescence-intensity measurements (*P* < 0.001; Fig. 3d). The expression of N-cadherin was inversely associated with that of E-cadherin in the MAWBP&D and Vector groups (*P* < 0.05; Fig. 3a, b, d). However, the expression of PGC showed the same trend as E-cadherin expression: PGC expression was increased relative to control in the MAWBP&D group and was lowest in the Vector group (*P* < 0.001; Fig. 4). Lastly, the expression of Snail protein was weakest in the MAWBP&D group and increased in the Vector group (*P* < 0.05; Fig. 4a, c). We found that cells in the MAWBP&D group were well organized and appeared to exhibit polarity, whereas the cells in the control group were disorganized (Fig. 3d, Fig. 4c).

We also measured AKP activity to further analyze the differentiation level of various transfected cells. The AKP levels were the following (in U/g protein): MAWD group, 77.3 ± 5.8; MAWBP group, 74.8 ± 3.9; MAWBP&D group, 51.6 ± 12.1; and Vector group, 91.9 ± 3.5. AKP activity was lowest in the MAWBP&D group and highest in the control group (*P* < 0.001; Fig. 3c). Collectively, the aforementioned results suggest that MAWD and MAWBP induce the differentiation of GC cells.

### Potential MAWD and MAWBP protein-protein interaction (PPI) networks

**Fig. 5:**
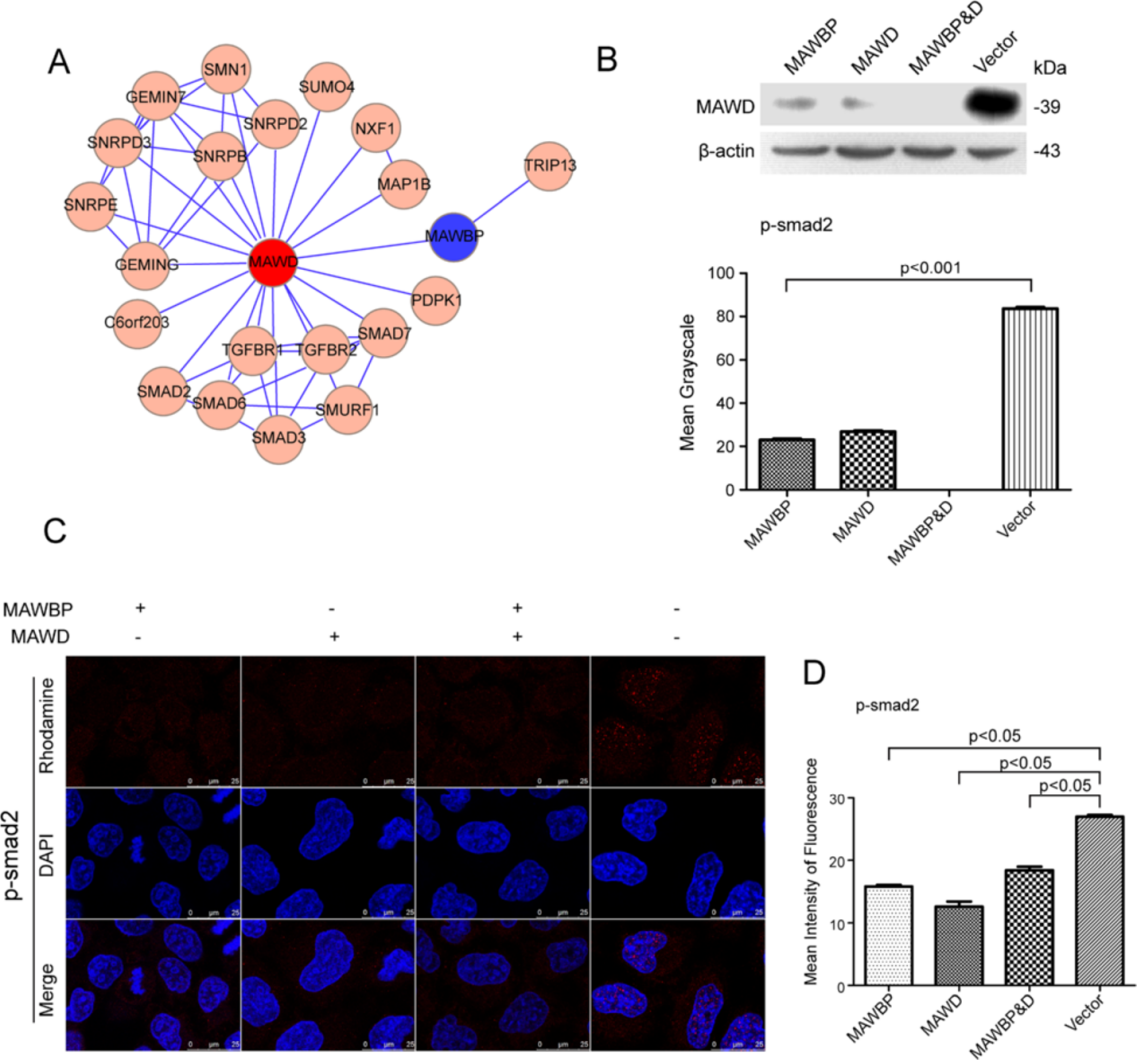
MAWBP and MAWD influence the expression of differentiation-related proteins in GC cells through TGF-beta signaling. (**a**) Prediction of MAWBP and MAWD protein-protein interaction networks. A schematic showing the protein interaction network of MAWMP and MAWD, retrieved from http://www.hprd.org/. (**b**) Western blotting results showed that the p-Smad2 level was lowest in the MAWBP&D group. (**c**-**d**) Confocal analysis revealed that the nuclear-translocation capacity of *p*-Smad2 was low in the MAWBP and MAWD overexpression groups and highest in the Vector group

PPI networks were identified here and these provided complementary evidence to our previous proteomics studies on MAWD and MAWBP interactions. MAWD interacted with proteins related to the TGF-beta signaling pathway, including TGF-beta and Smad2 (Fig. 5a).

### Coexpression of MAWD and MAWBP influences the TGF-beta signaling pathway

Western blotting analysis performed on the transfected cells revealed that p-Smad2 levels were lowest in the MAWBP&D group and highest in the Vector group (Fig. 5b). Furthermore, the nuclear-translocation capacity of p-Smad2 was lowest in the MAWBP&D group, as shown by confocal microscopy (Fig. 5c), and the mean fluorescence intensity of *p*-Smad2 was highest in the Vector group (*P* < 0.05; Fig. 5d). These results indicate that the MAWBP-MAWD complex could effectively suppress TGF-beta signaling by inhibiting downstream phosphorylation.

### Overexpression of MAWD and MAWBP affects the tumorigenicity of GC cells

**Fig. 6:**
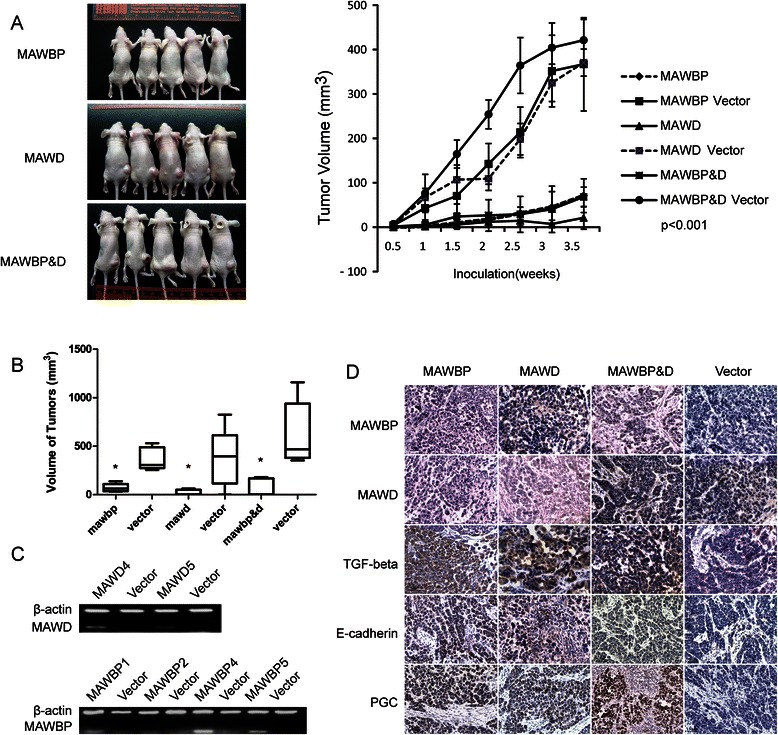
Co-overexpression of MAWBP and MAWD influences the tumorigenicity of GC cells. (**a**-**b**) Tumors induced by cells of the MAWBP, MAWD, and MAWBP&D groups were smaller than those induced by cells of the control group (*P* < 0.001). (**c**) RT-PCR analysis revealed that MAWBP and MAWD were overexpressed in xenografts derived from cells transfected with MAWBP and MAWD. (**d**) IHC analysis for comparison of MAWBP, MAWD, TGF-beta, E-cadherin, and PGC expression in different xenograft tumors.

The results of *in vivo* experiments showed that tumor growth was slower in nude mice injected with cells of the MAWD, MAWBP, and MAWBP&D groups than in mice injected with cells of the control group (Fig. 6a). Tumor growth was clearly slower after injection with cells of the MAWD and MAWBP groups as compared to that after injection of the Vector-group cells (*P* < 0.001; Fig. 6a). Moreover, the tumor volume in the MAWD and MAWBP overexpression groups was lower than that in the Vector group (*P* < 0.001; Fig. 6b). RT-PCR results showed that MAWD and MAWBP were overexpressed in xenografts derived from cells transfected with MAWD and MAWBP (Fig. 6c), and this was confirmed by the immunostaining results (Fig. 6d). We also used IHC to evaluate the expression of TGF-beta, E-cadherin, and PGC in excised xenograft tumors; the proteins showed varied expression in distinct groups but the expression was higher in the MAWD and MAWBP overexpression groups than in other groups (Fig. 6d). In Additional file 1, we present a model to illustrate the molecular functions of MAWD and MAWBP in the differentiation of GC cells.

## Discussion

In this study, we systematically confirmed the correlation between the overexpression of MAWD and MAWBP and differentiation in GC tissues and cell lines. More importantly, we found that the coexpression of MAWD and MAWBP correlated with the expression of E-cadherin and PGC, which are differentiation-related factors in gastric cells. Furthermore, the expression of N-cadherin, Snail, and p-Smad2 was inversely associated with that of E-cadherin and PGC, and overexpression of MAWD and MAWBP reduced the nuclear translocation of Smad2 by attenuating its phosphorylation.

Previously, we reported proteomic data acquired from screening GC protein profiles, including those of MAWD and MAWBP, and we showed that these proteins can form a complex [8]. Thus, combined analysis of MAWD and MAWBP expression should provide useful information for uncovering the roles of these proteins in GC. We verified the expression of these 2 potential GC-related proteins in several GC tissue samples by means of TMA and IHC analyses. We found that MAWD and MAWBP were expressed at low levels in GC tissues, and that the expression of TGF-beta was also substantially decreased in GC; the expression levels of all 3 of these proteins were correlated. These results agree with previous observations. The proteins were also related to GC differentiation grade and patient prognosis. The survival times of patients who expressed high levels of MAWD and MAWBP were longer than those of patients who expressed these proteins at low levels.

Next, we analyzed the relationship between MAWD and MAWBP expression and differentiation in GC tissues by examining the differentiation-related proteins E-cadherin and PGC. E-cadherin plays a major role in cell-cell interactions, and a reduction in E-cadherin expression is correlated with de-differentiation, invasiveness, and metastatic activity of carcinoma cells [22]. PGC is an aspartic protease produced mainly by the gastric mucosa [23], and the expression of PGC is used as a biomarker for the gastric mucosa. Moreover, a change in PGC expression might reflect gastric-cell differentiation [24], and the levels of E-cadherin and PGC can reflect the severity of gastric lesions or gastric-cell differentiation [25, 26]. Here, we detected E-cadherin and PGC expression in the TMA, and we found that whereas the expression of MAWD, MAWBP, and TGF-beta was clearly correlated with that of E-cadherin, PGC expression was correlated with MAWD expression. These results provided evidence indicating that the expression of MAWD and MAWBP is closely related with the differentiation of GC.

We used PPI bioinformatic predictions to extract all available human proteins that are related to MAWD and MAWBP, and we described their global properties. PPI bioinformatic predictions could provide complementary evidence for genome-wide experimental studies. The function annotation of MAWD-interacting proteins indicated the potential involvement of MAWD and MAWBP in TGF-beta signaling.

We next evaluated the relationship between MAWD and MAWBP expression and differentiation in GC cells. We constructed eukaryotic expression vectors of MAWD and MAWBP, transfected them alone or together into SGC7901 cells, and examined the expression of the differentiation-related proteins E-cadherin and PGC in various transfected clones. We found that E-cadherin and PGC were strongly expressed in cells cotransfected with MAWBP and MAWD. Confocal analysis revealed that the cells in the MAWBP&D group were well organized and appeared to exhibit polarity, whereas the cells in the control group were disorganized. Furthermore, the results of *in vivo* xperiments showed that tumor growth was slower in nude mice injected with cells of the MAWD, MAWBP, and MAWBP&D groups as compared with that in mice injected with cells of the control group. E-cadherin and PGC were also expressed at the highest level in the xenograft tumors of the MAWBP&D group. These results indicate that the cells in the MAWBP&D group were differentiated to a greater extent than the cells in the other groups.

A malignant gastric tumor cell might also produce a particular isozyme of an enzyme, as illustrated most clearly in the case of AKP production. AKP activity was reported to be inversely proportional to GC cell differentiation [27]. We measured AKP activity in various transfected cells and found that the activity was lowest in the MAWBP&D-transfected clones, but highest in the Vector group. These results also suggested that the degree of differentiation was highest in the MAWBP&D clones. Thus, overexpression of both MAWD and MAWBP induced GC differentiation.

E-cadherin is expressed by most epithelial tissues, and certain proteins expressed in cancer cells are also related to E-cadherin, such as Snail, Smad2, and Smad3. Conversely, N-cadherin is an adhesion molecule that is typically expressed by mesenchymal cells. The loss of E-cadherin expression and the gain of N-cadherin expression in cancer cells, occasionally referred to as “the cadherin switch,” are functionally significant in cancer progression [28]. Furthermore, the molecule Snail could be related to E-cadherin because Snail can bind to specific DNA sequences called E-boxes present in the E-cadherin promoter and repress transcription [29]. Thus, we measured the expression levels of N-cadherin and Snail in the transfected GC cells; whereas E-cadherin was downregulated and N-cadherin was upregulated in control SGC7901 cells, E-cadherin was upregulated and N-cadherin was downregulated in MAWBP&D-cotransfected SGC7901 cells. Moreover, in MAWBP&D-cotransfected cells, we also noted a reduction in the expression of Snail, a molecule that can be induced by TGF-beta stimulation [30]. MAWD was found to recruit Smad7 and form a complex that inhibited TGF-beta signaling. Therefore, we evaluated TGF-beta activity in different transfected cells by examining the phosphorylation level and nuclear translocation of Smad2. We found that the p-Smad2 level was lowest in the cells of the MAWBP&D group but highest in the control-group cells, and that the nuclear-translocation ability of p-Smad2 was also weakest in the cotransfected cells. Our results suggest that MAWD cooperates with MAWBP to inhibit the TGF-beta signaling pathway, which decreases Snail expression and increases E-cadherin expression; this, in turn, results in the well-differentiated characteristics of MAWBP&D-cotransfected cells.

In conclusion, MAWD and MAWBP were downregulated in GC tissues and associated with the differentiation grade of these tissues. Low MAWD and MAWBP expression levels were associated with poor survival of patients. The results of *in vitro* and *in vivo* experiments demonstrated that the co-overexpression of MAWD and MAWBP induced GC differentiation. Lastly, detection of p-Smad2 levels and its nuclear translocation revealed that MAWD and MAWBP induced the expression of differentiation-related proteins by modulating TGF-beta signaling in GC cells.

## Conclusions

We discovered that MAWD and MAWBP, which were downregulated in GC tissues, were associated with differentiation in GC tissues. Coexpression of MAWD and MAWBP was correlated with the expression of the differentiation-related proteins E-cadherin and PGC in GC cells. The levels of Snail (a factor that negatively regulates E-cadherin expression), N-cadherin, and p-Smad2 were inversely correlated with E-cadherin levels in cells that coexpressed MAWD and MAWBP. The results of AKP assays confirmed that these MAWD/MAWBP-coexpressing cells were differentiated to a greater extent as compared to control cells. Coexpression of MAWD and MAWBP also influenced the expression of E-cadherin and PGC *in vivo*. Our results suggest that the MAWD and MAWBP might induce the expression of differentiation-related proteins through the modulation of TGF-beta signaling in GC cells (Additional file 1).

## Additional file

Additional file 1: Figure S1. A model illustrating the molecular functions of MAWBP and MAWD in GC. The presence of MAWBP enhances the inhibitory effect of MAWD on the TGF-beta signaling pathway. The MAWD-MAWBP complex inhibits the phosphorylation and nuclear translocation of Smads, which influences the expression of downstream genes, as shown by, for example, the downregulated expression of the transcription factor Snail. Snail does not efficiently bind to the E-box upstream of the E-cadherin gene, and thus the expression of E-cadherin is increased. This pathway influences the differentiation of GC cells. (TIFF 945 kb)

## Abbreviations

MAWD: Mitogen-activated protein kinase activator with WD40 repeats
MAWBP: MAWD-binding protein
GC: Gastric cancer
TGF-beta: Transforming growth factor-beta
PGC: Pepsinogen C
TMA: Tissue microarray
IHC: Immunohistochemistry
AKP: Alkaline phosphatase
EGFR: Epidermal growth factor receptor
PPI: Protein-protein interaction
RT-PCR: Reverse-transcription PCR
SPSS: Statistic package for social science
