# Supplementary figures and images for: Mitogen-activated protein kinase activator with WD40 repeats (MAWD) and MAWD-binding protein induce cell differentiation in gastric cancer
Source: BMC Cancer. 2015 Sep 15;15:637. doi: 10.1186/s12885-015-1637-7 (PMC4572691; doi:10.1186/s12885-015-1637-7)

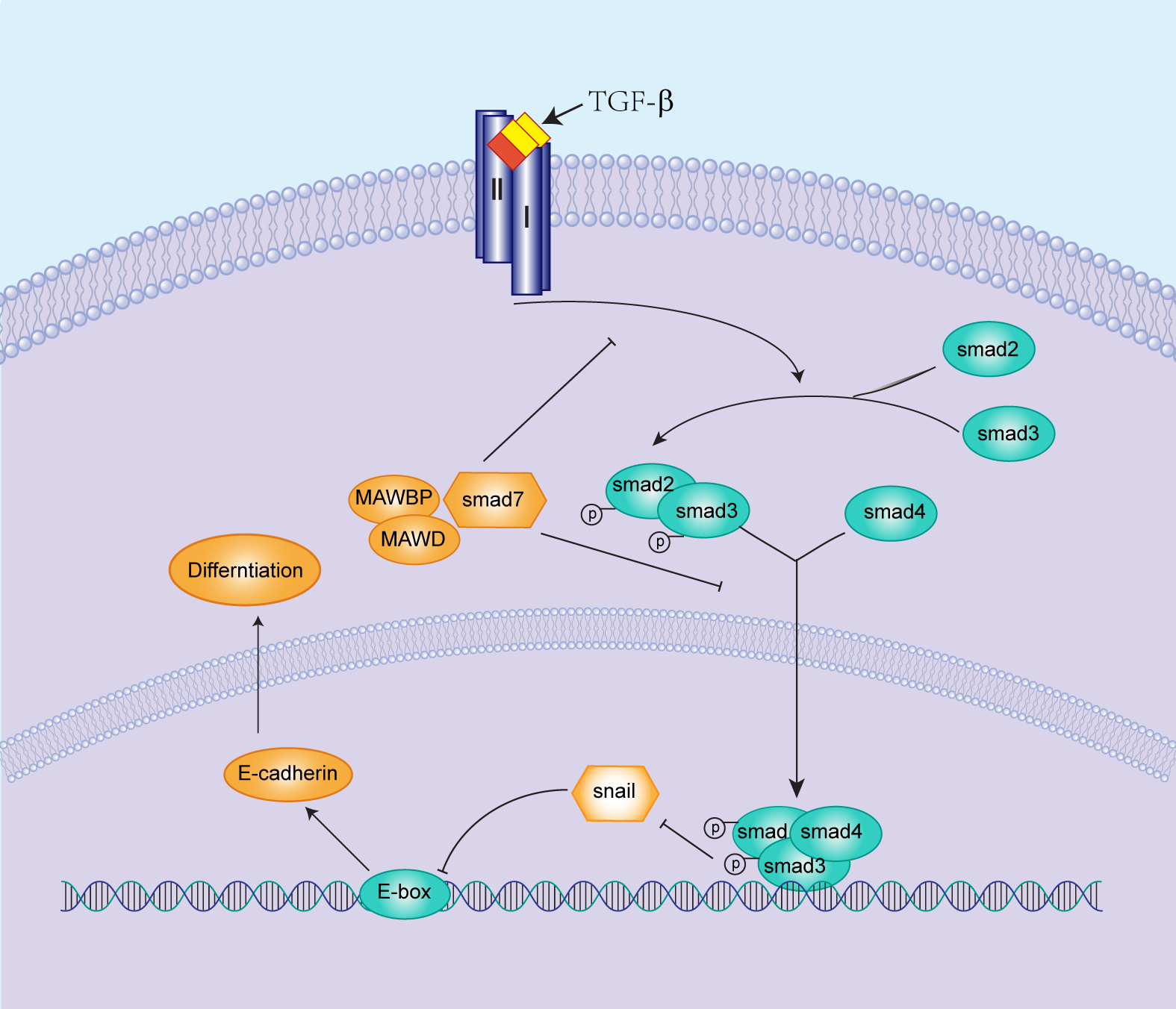

Supplement: Additional file 1: Figure S1. — A model illustrating the molecular functions of MAWBP and MAWD in GC. The presence of MAWBP enhances the inhibitory effect of MAWD on the TGF-beta signaling pathway. The MAWD-MAWBP complex inhibits the phosphorylation and nuclear translocation of Smads, which influences the expression of downstream genes, as shown by, for example, the downregulated expression of the transcription factor Snail. Snail does not efficiently bind to the E-box upstream of the E-cadherin gene, and thus the expression of E-cadherin is increased. This pathway influences the differentiation of GC cells. (TIFF 945 kb) [file 12885_2015_1637_MOESM1_ESM.tiff]
